# Structure of importin-α bound to a non-classical nuclear localization signal of the influenza A virus nucleoprotein

**DOI:** 10.1038/srep15055

**Published:** 2015-10-12

**Authors:** Ryohei Nakada, Hidemi Hirano, Yoshiyuki Matsuura

**Affiliations:** 1 Division of Biological Science Graduate School of Science Nagoya University Japan; 2 Structural Biology Research Center, Graduate School of Science, Nagoya University, Japan

## Abstract

A non-classical nuclear localization signal (ncNLS) of influenza A virus nucleoprotein (NP) is critical for nuclear import of viral genomic RNAs that transcribe and replicate in the nucleus of infected cells. Here we report a 2.3 Å resolution crystal structure of mouse importin-α1 in complex with NP ncNLS. The structure reveals that NP ncNLS binds specifically and exclusively to the minor NLS-binding site of importin-α. Structural and functional analyses identify key binding pockets on importin-α as potential targets for antiviral drug development. Unlike many other NLSs, NP ncNLS binds to the NLS-binding domain of importin-α weakly with micromolar affinity. These results suggest that a modest inhibitor with low affinity to importin-α could have anti-influenza activity with minimal cytotoxicity.

Influenza A virus is a serious pathogen with the capacity to cause seasonal epidemics and high mortality pandemics^1^. Currently, all of the clinically approved anti-influenza drugs target the viral proteins^1^. However, due to the high mutation rate and frequent genetic reassortment of influenza viruses, rapid emergence of drug resistant strains is virtually inevitable. In this context, targeting the host proteins exploited by the virus during its replication cycle is an attractive strategy to reduce the development of drug resistance^2^,^3^,^4^. Because influenza viruses transcribe and replicate their genome in the nucleus of the host cell, the viral genome needs to be imported into the nucleus early in infection, and the progeny viral genome needs to be exported from the nucleus to cytoplasm at late time points of infection. Thus the host nuclear trafficking machinery is essential for viral replication^5^ and represents a set of potential target for small molecule intervention.

The genome of the influenza A virus is segmented into eight negative-sense RNA molecules, each of which is associated with the vial RNA polymerase subunits (PA, PB1, and PB2) and many molecules of nucleoprotein (NP) to form eight viral ribonucleoprotein (vRNP) complexes. The nuclear import of vRNPs is mediated by importin-α:β heterodimer^6^. Importin-α:β mediates nuclear import of numerous proteins bearing a nuclear localization signal (NLS)^7^,^8^,^9^,^10^,^11^,^12^. Importin-α binds directly to the NLS and then recruits importin-β via importin-α′s importin-β-binding (IBB) domain^13^,^14^ to form a ternary complex that can dock at and move through nuclear pore complexes. The majority of cargoes recognized by importin-α contain classical NLSs (cNLSs) that fall into two distinct classes termed monopartite NLSs, containing a single cluster of basic amino acids, and bipartite NLSs, comprising two basic clusters separated by a linker of variable length^15^. The NLS-binding domain of importin-α is an elongated superhelical domain built from 10 tandem ARM repeats^16^,^17^. The cNLSs bind along the groove on the inner concave surface of the ARM repeat domain^16^,^18^,^19^. The monopartite cNLSs bind to the major NLS-binding site located on ARM repeats 2–4, whereas the bipartite cNLSs bind to both this major site and the minor NLS-binding site located on ARM repeats 6–8. Recently, novel NLSs that bind specifically and exclusively to the minor site of importin-α have been identified^20^,^21^,^22^,^23^, although a bioinformatic analysis indicated that the minor-site specific NLSs are much less prevalent than the cNLSs^23^.

The NP protein encodes an NLS at its amino terminus that has been shown to function as the major signal necessary for the nuclear import of vRNPs^24^,^25^,^26^. This NLS is termed non-classical NLS (ncNLS) because it does not compete with the classical SV40 NLS (the representative monopartite cNLS) for the binding to importin-α^26^. The NP ncNLS is also important for nuclear import of newly synthesized free NP proteins that are required for assembly of progeny vRNPs^24^,^25^,^26^. Specifically, three lines of previously reported biochemical and cell biological data provided strong support for the proposal that the ncNLS is required for nuclear import of the NP protein via direct binding to importin-α, and that the ncNLS is functional in the context of the intact, full-length NP. First, substitution of the basic residues of ncNLS (i.e., K7 and R8) with alanine abolished nuclear import of full-length NP^25^,^26^, and also drastically weakened the binding of importin-α to full-length NP in GST pull-down assay^26^. Secondly, in nuclear import assay using digitonin-permeabilized cells, nuclear import of NP and vRNPs was inhibited by ncNLS peptide but not by SV40 NLS^26^. Thirdly, in GST pull-down assay, the binding of importin-α to full-length NP was inhibited by ncNLS peptide but not by SV40 NLS^26^. In the X-ray crystal structures of NP^27^,^28^, the N-terminus of NP is disordered and protrudes out of the folded domain, indicating that the ncNLS of free NP is accessible for importin-α binding. The NP ncNLS is also exposed to solvent in the structural models of vRNP based on electron microsopy (EM)^29^,^30^, and it seems likely that multiple importin-α:β complexes are recruited to vRNP via binding to the ncNLS of NP. Although it has been proposed that there is another NLS (bipartite cNLS) in the middle of the NP protein^31^,^32^, the NP crystal structures suggest that this putative NLS motif would not bind importin-α unless NP unfolds^27^,^28^, and functionality of this putative NLS has been questioned by mutational analyses^26^.

The fact that the major NLS of vRNPs is of a non-classical type suggests that targeting the host importin-α could be a viable strategy to develop novel antiviral compounds to inhibit nuclear import of vRNPs and free NP without interfering with nuclear import of many (if not all) of the host proteins. Here we report the crystal structure of importin-α bound to NP ncNLS. Structural and functional analyses identify key binding pockets on importin-α and suggest that a modest inhibitor that binds to these sites with micromolar affinity could be an effective anti-influenza drug with minimal cytotoxicity.

## Results and Discussion

### Crystal structure of importin-α bound to NP ncNLS

**Figure 1:**
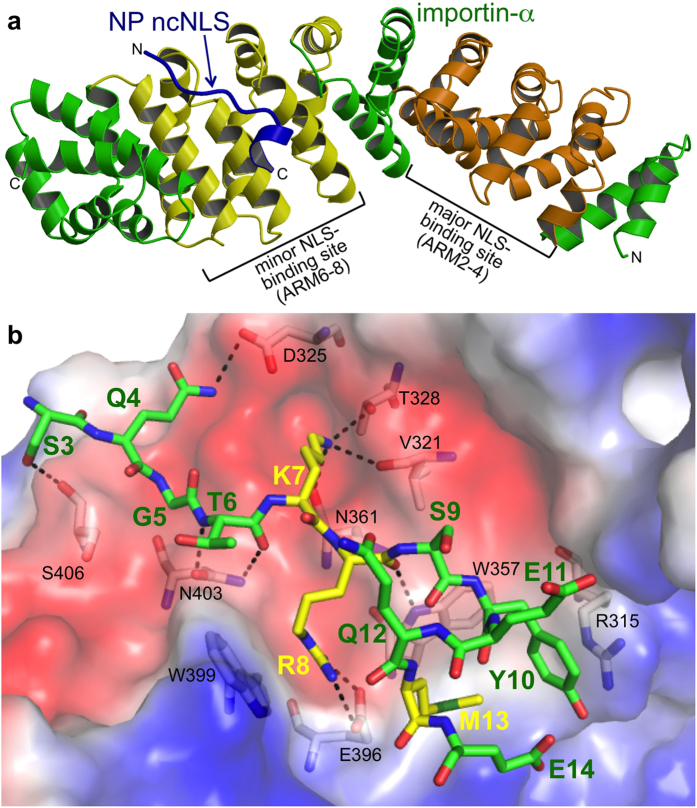
NP ncNLS binds to the minor-NLS binding site of importin-α. (**a**) Ribbon representation of the structure of importin-α ARM repeat domain (green, except for the major and minor NLS-binding sites that are highlighted in orange and yellow, respectively) in complex with NP ncNLS (blue). (**b**) Close-up view of the interactions involving NP ncNLS (stick representation with green carbons, except for K7, R8, and M13 that are highlighted in stick representation with yellow carbons) with key residues (stick representation with white carbons) under the transparent surface (colored by electrostatic potential: blue, positive; red, negative; white, neutral) of importin-α. Dashed lines indicate hydrogen bonds or salt bridges. See also Supplementary Movie 1.

**Figure 2:**
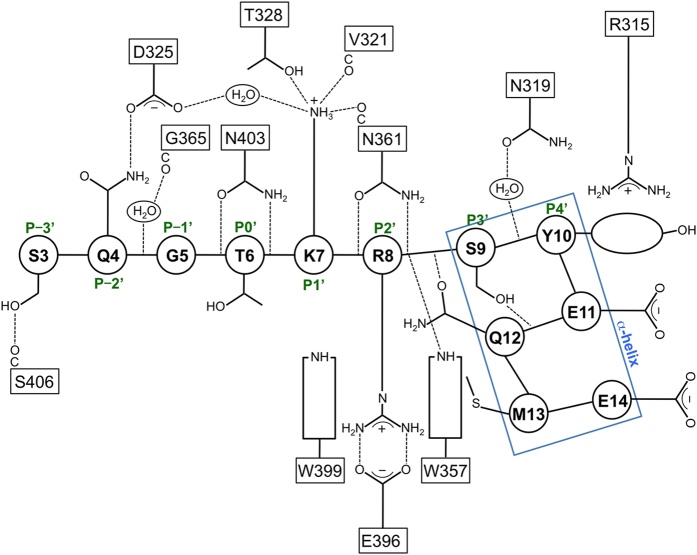
Schematic illustration of the interactions between importin-α and NP ncNLS at the minor NLS-binding site. The residue numbers of NP ncNLS are shown in circles, whereas the residue numbers of importin-α are shown in rectangles. Dashed lines represent hydrogen bonds or salt bridges. The minor-site positions P−3′ to P4′ are indicated.

To elucidate the structural basis for how NP ncNLS binds to importin-α to ultimately develop novel therapeutic strategies, we determined a 2.3 Å resolution crystal structure of the NLS-binding ARM-repeat domain of mouse importin-α1 in complex with NP ncNLS by molecular replacement (Fig. 1a; Supplementary Table 1). Electron density corresponding to NP residues 3–14 was unambiguously identified at the minor NLS-binding site (Supplementary Fig. 1), in agreement with previous observations that NP does not compete with SV40 NLS^26^ and binds to the C-terminal ARM repeats of importin-α^33^. The structure showed that the NP ncNLS binds to the minor NLS-binding site through a network of interactions (Figs 1b and 2; see also Supplementary Movie 1) in a manner analogous to that reported for some of the minor-site specific NLSs such as the NLS of RNA helicase II/Guα^23^. The N-terminal region (residues 3–8) of the NP ncNLS adopts an extended conformation and occupies the P-3′-P2′ positions, whereas the C-terminal region (residues 9–14) forms an α-helical turn and packs against the P4′ position at the minor NLS-binding site. The main chain of the N-terminal region of ncNLS is oriented and anchored on importin-α through multiple hydrogen bonds with the exposed asparagine/tryptophan ladder along the NLS-binding groove. The side chains of ncNLS make mainly polar interactions with importin-α, supplemented with hydrophobic and van der Waals interactions. The NP residues S3 and Q4 at the P-3′ and P-2′ positions form hydrogen bonds with S406 and D325, respectively. The basic NP residues K7 and R8 occupy the core P1′ and P2′ positions. K7 forms hydrogen bonds with V321, T328, and N361. R8 forms a salt bridge with E396 and also makes a cation-π interaction with the indole ring of W399. Another cation-π interaction is made between the phenolic side chain of Y10 at the P4′ position and R315. Juxtaposed to Y10, the hydrophobic side chain of NP residue M13 makes an intimate contact with the nonpolar patch on the indole ring of W357. The side chains of the NP residues S9 and Q12 are involved in intramolecular hydrogen bonds with NP main chain atoms and may stabilize the α-helical conformation of the C-terminal region of ncNLS.

### Mutational analyses of the interactions between importin-α and NP ncNLS

**Figure 3:**
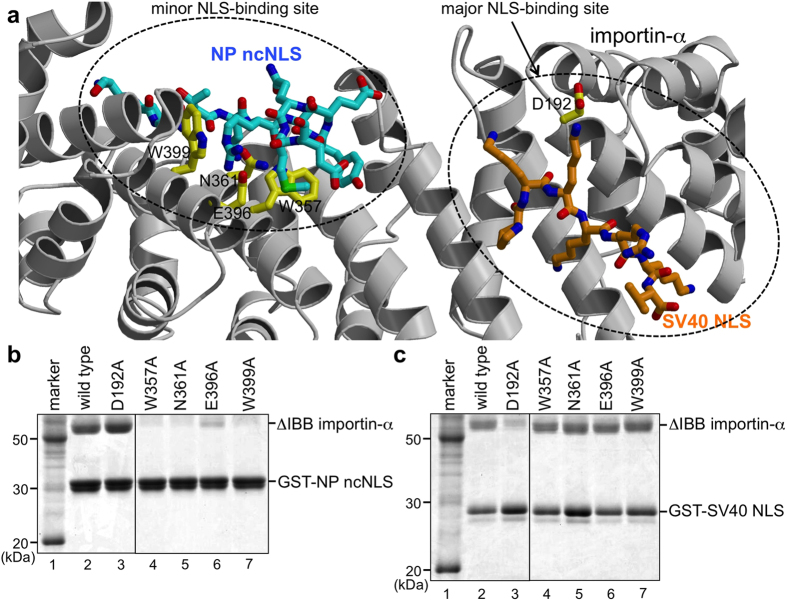
Mutational analyses verified that NP ncNLS and SV40 NLS bind to distinct sites on importin-α. Shown in (**a**) is the position of SV40 NLS (stick representation with orange carbons) at the major NLS-binding site of importin-α when the crystal structure of importin-α-SV40 NLS complex (PDB code, 1EJL)^19^ is superposed on top of the crystal structure of importin-α (ribbon representation in light gray) bound to NP ncNLS (stick representation with cyan carbons). The side chains of the residues of importin-α substituted with alanine in the functional analyses are highlighted in stick representation with yellow carbons. (**b,c**) GST pull-down assays using mutants of importin-α. Immobilized GST-NP ncNLS (**b**) or GST-SV40 NLS (**c**) was incubated with ΔIBB importin-α (wild-type or mutant).

Mutagenesis of importin-α verified that NP ncNLS binds to the minor-NLS binding site but not to the major NLS-binding site of importin-α in solution (Fig. 3). In GST pull-down assays, substitution of W357 or N361 or E396 or W399 at the minor NLS-binding site with alanine drastically reduced the binding to NP ncNLS, whereas the same alanine substitutions did not affect the binding to SV40 NLS (Fig. 3b). In contrast, alanine substitution of D192 at the major NLS-binding site reduced the binding to SV40 NLS but did not affect the binding to NP ncNLS (Fig. 3c). These results confirm that NP ncNLS and SV40 NLS bind to distinct sites of importin-α in solution.

**Figure 4:**
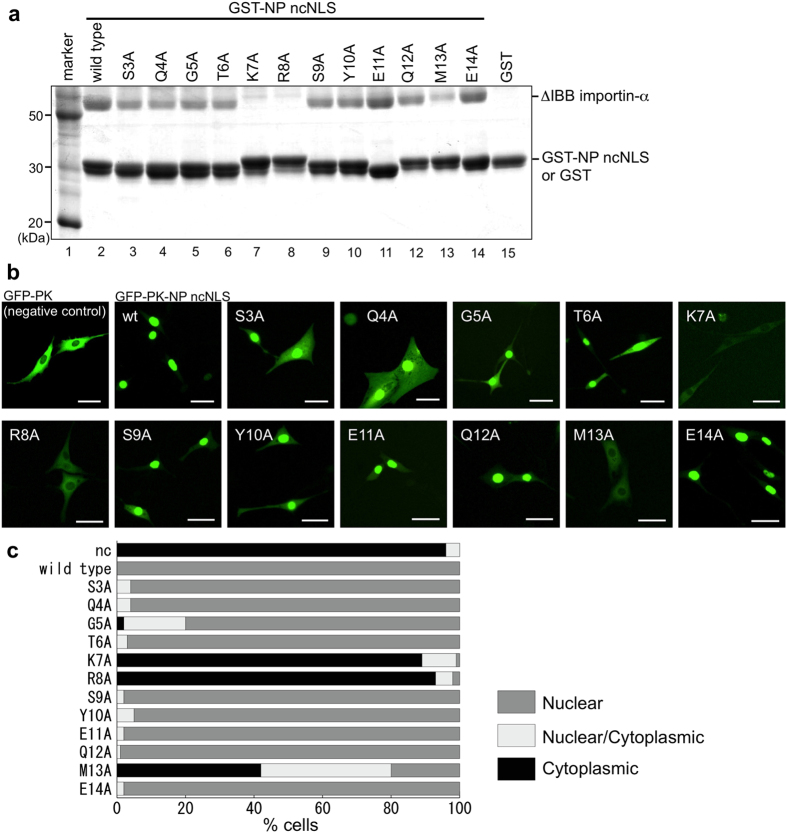
Alanine scanning mutational analyses of NP ncNLS. (**a**) Mutational analyses *in vitro*. GST pull-down assay showed that K7, R8, and M13 of NP ncNLS are crucial for importin-α binding. Immobilized GST-NP ncNLS (wild-type or mutant) or GST (negative control) was incubated with ΔIBB importin-α. Virtually no binding was seen to GST alone. (**b,c**) Mutational analyses *in vivo*. GFP-PK-NP ncNLS (wild-type or mutant) or GFP-PK (negative control) was expressed in NIH3T3 cells, and its subcellular localization was analyzed by fluorescence microscopy. (**b**) Representative images of the cells. Scale bar, 40 μm. (**c**) Localization of GFP-PK-NP ncNLS (wild-type or mutant) or GFP-PK (nc, negative control) was scored in 100 cells as follows: Nuclear (gray), Nuclear/Cytoplasmic (comparable intensity in nucleus and cytoplasm; light gray), or Cytoplasmic (black).

Alanine-scanning mutagenesis of NP ncNLS showed that not all ncNLS residues are equally important for importin-α binding (Fig. 4a) and nuclear import (Fig. 4b,c). Alanine substitution of NP residues K7 or R8 dramatically reduced importin-α binding and fully abolished the NLS activity. The amino acid substitution M13A was not as effective as K7A or R8A but still reduced importin-α binding and the NLS activity appreciably. The other alanine substitutions of NP ncNLS residues were much less effective. Thus a small subset of NP ncNLS residues, namely K7, R8, and M13, make key contributions in nuclear import.

### Regulation by phosphorylation of NP

The structural basis for NP ncNLS-importin-α interactions also provides a rationale to understand why nuclear trafficking of vRNPs can be regulated by phosphorylation/dephosphorylation of NP. In the late stage of influenza virus infection, the progeny vRNPs are exported from the nucleus to cytoplasm, and posttranslational modifications of NP have been proposed to be one of the mechanisms to prevent the exported vRNPs from going back to the nucleus. It is known that some of the NP ncNLS residues (S3, S9, and Y10) are phosphorylated at late stage of infection, leading to reduction of the NLS activity^34^,^35^. Our structure suggests that phosphorylation of S3 and Y10 would disrupt the NP-importin-α interactions at the edges of the ncNLS binding site. Phosphorylation of S9 could disrupt the C-terminal α-helical conformation of the ncNLS and thereby indirectly affect importin-α-binding. Alternatively, it is also conceivable that the phosphorylation of these NP residues recruits as yet unidentified binding partner(s) to mask the ncNLS.

### Implications for antiviral drug development

**Figure 5:**
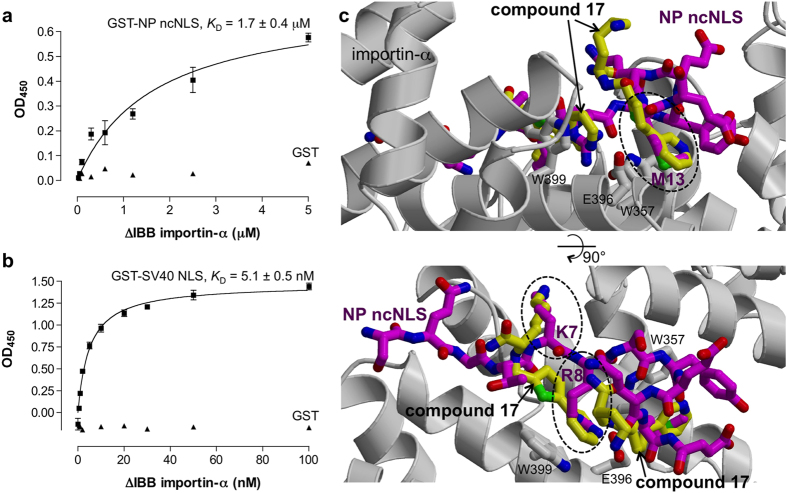
Implications for drug development. (**a,b**) Solid phase binding assay showed that NP ncNLS binds to ΔIBB importin-α more weakly than SV40 NLS. The *K*_D_ values represent the best fit value ± standard error. Each assay was performed in triplicate. Virtually no binding was seen to GST alone (triangle, negative control). (**c**) Two orthogonal views of the superposition of NP ncNLS (stick representation with magenta carbons) and a minor-site specific inhibitor (“compound 17”; stick representation with yellow carbons; PDB code, 4U5V)^43^ bound to importin-α (light gray). The compound 17 clashes with the three crucial residues (K7, R8, and M13) of NP ncNLS, which are marked by dashed ellipses.

An immediate suggestion from our structure is that one way to develop antiviral drugs would be to design small-molecule inhibitors that bind specifically to the minor NLS-binding site of importin-α. The binding pockets for the key residues (K7, R8, and M13) of NP are particularly attractive as the target binding sites for the inhibitors to block the NP binding effectively. The inhibitors directed against the ncNLS-binding site need to have sufficiently high affinity for importin-α to compete with NP. Interestingly, by using a solid phase binding assay, we found that NP ncNLS binds to the NLS-binding domain (ΔIBB importin-α) with micromolar affinity (*K*_D_ = 1.7 μM; Fig. 5a), which is two orders of magnitude weaker than the affinity of SV40 NLS to ΔIBB importin-α (*K*_D_ = 5.1 nM; Fig. 5b). The *K*_D_ value measured for SV40 NLS is comparable to the affinities observed previously^23^,^36^,^37^,^38^,^39^,^40^,^41^. Similarly to SV40 NLS, many cNLSs have affinity to ΔIBB importin-α in the 1–10 nM range, and the micromolar affinity of NP ncNLS is at the lower end of the affinity range suggested for functional NLSs (~1 nM to 1 μM)^23^,^36^,^37^,^38^,^39^,^40^,^41^,^42^. This suggests that small-molecule inhibitors with only micromolar affinity could block the binding of NP ncNLS without competing effectively with many other NLSs. It is also noteworthy that, even if the inhibitors have affinity similar to that of a subset of host NLSs and directly compete with those NLSs to bind the same site, it does not necessarily mean that the inhibitors are seriously toxic to the host. Given the acute nature of influenza infection, the therapy needs to be only short duration. The short duration of therapy may not be harmful to the host, provided that the inhibitors bind to host importin-α reversibly with only micromolar affinity and hence can be easily excreted from the host soon after the therapy. We therefore propose that optimization of the binding affinity would be an important strategy to minimize side effects of the inhibitors.

Recently, Holvey *et al.* published small-molecule compounds that bind specifically but weakly (*K*_D_ = ~ 1–10 mM) to the minor NLS-binding site of importin-α^43^. Among the minor-site specific compounds developed by Holvey *et al.*, the “compound 17” has the highest affinity (*K*_D_ = 0.9 mM)^43^. In the crystal structure of importin-α bound to this compound, two molecules of the compound bound to the minor NLS-binding site^43^. Strikingly, the compound bound to the binding pockets for the three critical residues (K7, R8, and M13) of NP ncNLS: one molecule of the compound bound to the binding pockets P1′ and P2′ (the binding pockets for K7 and R8 of NP; marked by dashed ellipses in the lower panel of Fig. 5c), and another molecule of the compound bound to the binding pocket for M13 of NP (marked by a dashed ellipse in the upper panel of Fig. 5c). Although the affinity of the compound 17 is probably too low to inhibit the binding of NP ncNLS, the compound 17 could serve as a starting point to optimize the binding affinity to develop an effective inhibitor of nuclear import of influenza vRNPs and free NP. Because the two molecules of compound 17 bound to importin-α are in close proximity, an obvious strategy to increase the affinity of the compound would be to link the two molecules of the compound covalently. It would also be possible to increase the affinity of the compound by attaching functional groups that can form hydrogen bonds with nearby residues of importin-α.

We conclude that, although it is unclear why influenza NP protein has evolved to hijack only the minor NLS-binding site, potentially, the structural and biochemical properties of the interactions between host importin-α and influenza NP ncNLS could be exploited to develop novel antiviral drugs.

## Materials and Methods

### Protein expression and purification for crystallization

An expression plasmid for glutathione *S*-transferase (GST)-tagged ncNLS (ASQGTKRSYEQMET; residues 2–15) of the NP of influenza virus strain A/Puerto Rico/8/34 (H1N1) was constructed by ligating complementary oligo-DNAs encoding the ncNLS into *Bam*HI/*Xho*I sites of pGEX-TEV^44^. To prepare the armadillo-repeat domain of *Mus musculus* (mouse) importin-α1 (Gene name, Kpna2; UniProt code, P52293) in complex with NP ncNLS, un-tagged mouse importin-α1 (residues 72–498)^45^ was expressed in the *Escherichia coli* host strain BL21-CodonPlus(DE3)RIL (Stratagene) at 20 °C from pET15b (Novagen), whereas GST-NP ncNLS was expressed separately in the same strain at 25 °C from pGEX-TEV. After harvesting, the two sets of cells were mixed, suspended in buffer A [10 mM Tris-HCl pH 7.5, 150 mM NaCl, 1 mM EDTA, 2 mM DTT, 1 mM phenylmethylsulfonyl fluoride (PMSF), 0.2 mM 4-(2-aminoethyl) benzenesulfonyl fluoride hydrochloride (AEBSF)] and lysed by sonication on ice. All subsequent steps were performed at 4 °C. Tween20 was added to 0.1%, and the clarified lysates were incubated with glutathione-Sepharose 4B (GE Healthcare) for 4 h. After washing the beads extensively with buffer B (10 mM Tris-HCl pH 7.5, 150 mM NaCl, 2 mM DTT, 0.05% Tween20), the GST-tag was removed with His-TEV protease (20 μg/ml) overnight. The importin-α1(72–498)-NP ncNLS complex released from the resin was finally purified over Superdex200 (GE Healthcare) in buffer C (10 mM Tris-HCl pH 7.5, 150 mM NaCl, 2 mM DTT). The complex was concentrated to 28 mg/ml using a 3 kDa molecular weight cutoff Amicon Ultra centrifugal filter (Millipore).

### Crystallization and data collection

Crystals of mouse importin-α1(72–498)-NP ncNLS complex were readily obtained by hanging drop vapor diffusion method using a screening kit suitable for crystallization of protein-protein complexes^46^. The final crystals used for structure determination were grown at 20 °C by equilibrating a drop containing 2 μl of protein solution (5 mg/ml in buffer C) and 2 μl of a reservoir solution (0.1 M MES pH 6.5, and 22% PEG4000) against 0.5 ml of the reservoir solution in VDX plates (Hampton Research). Plate-shaped crystals grew to maximum dimensions of 0.2 × 0.2 × 0.03 mm in 2 weeks. Crystals were serially transferred to 0.1 M MES pH 6.5, 24% PEG4000, and 23% glycerol in four steps and were flash-cooled in liquid nitrogen. Preliminary X-ray diffraction experiments were performed at SPring-8 beamline BL26B2, and a 2.3 Å resolution data set used for final structure determination was collected at 100 K at Photon Factory beamline BL-1A (Tsukuba, Japan). The crystals had *H*32 symmetry (*a* = *b* = 110.25 Å, *c* = 204.36 Å ) with one complex in the asymmetric unit.

### Structure determination and refinement

Diffraction data were processed using MOSFLM and CCP4 programs^47^. The structure of importin-α1(72–498)-NP ncNLS complex was solved by molecular replacement using MOLREP^48^ using the structure of mouse importin-α1 in the importin-α1-nucleoplasmin NLS complex^42^ (PDB ID, 3UL1) as a search model. Refinement of the molecular replacement solution using REFMAC5 (ref. 49) reduced *R*_free_ from 48.5% to 38.5%, after which there was clear difference density corresponding to the NP ncNLS in the minor NLS-binding site. Iterative cycles of model building using COOT^50^ and refinement using REFMAC5 and PHENIX^51^ yielded a final model with an *R*_free_ of 24.6% (*R*_cryst_ 18.8%) that contained importin-α1 residues 76–105, 111–496, NP residues 3–14, and 80 water molecules. A TLSMD analysis^52^ was used to define TLS groups for the final cycles of refinement. MolProbity^53^ was used to validate the final structure. Structural figures were produced using Molscript^54^, Raster3D^55^, and PyMOL (https://www.pymol.org). Coordinates and structure factors have been deposited in the Protein Data Bank under accession code 4ZDU.

### Protein expression and purification for binding assays

GST-NP ncNLS, GST-SV40 NLS^40^, and His/S-ΔIBB importin-α1 (mouse, residues 70–529)^19^ were expressed from pGEX-TEV, pGEX-4T-1, and pET30a, respectively, in *E. coli* strain BL21-CodonPlus(DE3)RIL (Stratagene). GST-NLS fusion proteins were purified over Glutathione-sepharose 4B (GE Healthcare) and gel filtration over Superdex200 (GE Healthcare). His/S-ΔIBB importin-α1 was purified over Ni-NTA (Novagen) and gel filtration over Superdex200 (GE Healthcare). Mutants were created using the Quickchange system (Stratagene), and all constructs were verified by DNA sequencing.

### GST pull-down assay

GST pull-down assays were performed in buffer D (30 mM Tris-HCl pH 7.5, 150 mM NaCl, 0.2 mM AEBSF, 0.05% Tween20). GST-NLS fusion proteins (50 μg) were immobilized on 10 μl of packed Glutathione-sepharose 4B (GE Healthcare) beads and incubated with His/S-ΔIBB importin-α1 (17 μg) in 50 μl of binding buffer for 1 h at 4 °C. Beads were then spun down and washed twice with 1 ml of binding buffer. Bound proteins were subsequently eluted with SDS-sample buffer, and analyzed by SDS-PAGE and Coomassie staining.

### Microtiter-plate binding assay

Solid phase binding assays were carried out on Immulon 2HB microtiter plates (Dynex) essentially as described previously^23^,^40^,^41^,^56^,^57^. The plates were coated with 15 ng GST-NLS or GST per well overnight at 4 °C in buffer E (PBS, 2 mM DTT, and 0.2 mM PMSF). The plates were then washed three times with PBS and incubated overnight at 4 °C in buffer F (PBS, 0.1% Tween20, 3% BSA, 2 mM DTT, and 0.2 mM PMSF). Binding reactions were carried out overnight at 4 °C with 100 μl/well of the indicated concentrations of His/S-ΔIBB importin-α1 in buffer F. After binding, the plates were washed three times with buffer F without BSA, and proteins were cross-linked for 15 min at room temperature by incubation in 1 mg/ml 1-etyl-3-(3-dimethylaminopropyl)carbodiimide (EDC) in the same buffer. The plates were then washed for 20 min in PBS-T (PBS supplemented with 0.1% Tween20), 10 min in PBS-T containing 100 mM ethanolamine, and finally incubated for 10 min in PBS-T containing 3% BSA. The bound His/S-ΔIBB importin-α1 was detected by incubation with S-protein-horseradish peroxidase conjugate (Novagen) in buffer E containing 1% BSA and 0.1% Tween20. After 1 h at 4 °C, the plates were washed three times with PBS. Horseradish peroxidase substrate (3,3′,5,5′-tetramethylbenzidine, Vector Laboratories) was added for 10 min at room temperature and the reaction was stopped by the addition of an equal volume of 0.5 M H_2_SO_4_. The signal was determined at 450 nm with an ImmunoMini NJ-2300 plate reader (Nalge Nunc). Binding data were analyzed with GraphPad Prism (GraphPad Software) using nonlinear regression assuming one-site binding.

### Cell culture, transfection, and live cell imaging

An expression plasmid for GFP-Pyruvate kinase (PK)-NP ncNLS fusion protein was constructed by cloning a PCR-amplified DNA fragment encoding a linker (GSDYDIPTTENLYFQGS) and the NP ncNLS (ASQGTKRSYEQMET) into *Eco*RI/*Sal*I sites of pEGFP-PK^45^. The mouse fibroblast NIH3T3 cells were grown in Dulbecco’s Modified Eagle’s Medium (DMEM) high glucose (4.5 mg/ml) (Wako) supplemented with 10% new born calf serum (GIBCO) at 37 °C in an atmosphere containing 5% CO_2_. For live cell imaging, NIH3T3 cells were transfected with expression plasmids for GFP-PK-NP ncNLS using Lipofectamine 2000 Transfection Reagent (Invitrogen). After two days, the GFP-PK-NP ncNLS proteins expressed in the transfected cells were observed under an Olympus IX81-DSU spinning-disk confocal fluorescence microscope with an excitation filter (460–480 nm) and an emission filter (495–540 nm).

## Additional Information

**How to cite this article**: Nakada, R. *et al.* Structure of importin-α bound to a non-classical nuclear localization signal of the influenza A virus nucleoprotein. *Sci. Rep.* **5**, 15055; doi: 10.1038/srep15055 (2015).

## Supplementary Material

Supplementary Information

Supplementary Movie 1
