# Supplementary material for: Structure of importin-α bound to a non-classical nuclear localization signal of the influenza A virus nucleoprotein
Source: Sci Rep. 2015 Oct 12;5:15055. doi: 10.1038/srep15055 (PMC4601014; doi:10.1038/srep15055)
Supplement: Supplementary Information [file srep15055-s1.pdf]

Supplementary Information

Structure of importin-α bound to a non-classical nuclear localization signal of the influenza A virus nucleoprotein

Ryohei Nakada, Hidemi Hirano, and Yoshiyuki Matsuura

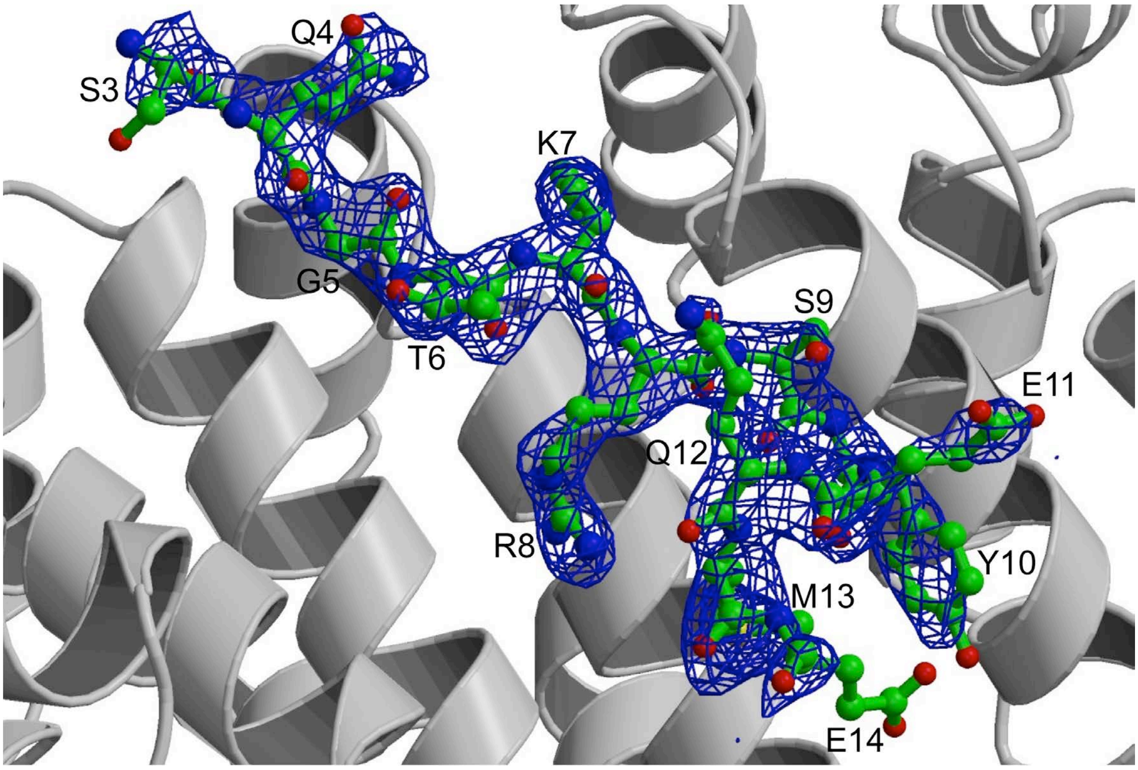

Supplementary Figure 1. The omit map covering NP ncNLS. The blue mesh shows the omit F_O–F_C electron density map contoured at 3.0σ after refinement, with NP ncNLS omitted. The density map is superposed onto the structure of NP ncNLS (ball-and-stick representation with green carbons) bound to the minor-NLS binding site of importin-α (ribbon representation in light gray).

Supplementary Table 1. Crystallographic data and refinement statistics

Data collection statistics	
Space group	*H*32
Unit cell dimensions	
a, b, c (Å)	110.25, 110.25, 204.36
α, β, γ (degree)	90.0, 90.0, 120.0
Resolution range (Å)[a]	31.83-2.30 (2.38-2.30)
Total observations[a]	197452 (19667)
Unique reflections[a]	21538 (2070)
Completeness (%)[a]	100.0 (100.0)
Multiplicity[a]	9.2 (9.5)
R_{merge} (%)[a]	9.9 (80.2)
Mean I/σ[a]	14.4 (3.3)
Mean I half-set correlation CC(1/2)	0.999 (0.955)
Refinement statistics	
Resolution range (Å)	31.83-2.30
R_{cryst}/R_{free} (%)	18.8/24.6
Total number of non-H atoms	
Protein	3252
Water	80
Average B-factors ($Å^2$)	
Protein (importin-α)	52.4
Protein (NP)	58.9
Water	51.1
r.m.s. deviation from ideality	
bond length (Å)	0.003
bond angles (degree)	0.808
Protein geometry[b]	
Rotamer outliers (%)	0
Ramachandran outliers (%)	0
Ramachandran favored (%)	98.8
Cβ deviations > 0.25Å	0
Residues with bad bonds (%)	0
Residues with bad angles (%)	0
PDB code	4ZDU

[a] Parentheses refer to final resolution shell

[b] MolProbity was used to analyze the structure.

Supplementary Movie 1. The movie illustrates the interactions between importin-α and NP ncNLS. Coloring is according to Figure 1b. The key residues mentioned in the text (K7, R8, and M13 of NP) are closed up one by one. The movie was created using PyMOL (https://www.pymol.org).
